# DeepNOG: fast and accurate protein orthologous group assignment

**DOI:** 10.1093/bioinformatics/btaa1051

**Published:** 2020-12-26

**Authors:** Roman Feldbauer, Lukas Gosch, Lukas Lüftinger, Patrick Hyden, Arthur Flexer, Thomas Rattei

**Affiliations:** btaa1051-aff1 Department of Microbiology and Ecosystem Science, University of Vienna, Vienna 1090, Austria; btaa1051-aff2 Ares Genetics GmbH, Vienna 1030, Austria; btaa1051-aff3 Institute of Computational Perception, Johannes Kepler University Linz, Linz 4040, Austria

## Abstract

**Motivation:**

Protein orthologous group databases are powerful tools for evolutionary analysis, functional annotation or metabolic pathway modeling across lineages. Sequences are typically assigned to orthologous groups with alignment-based methods, such as profile hidden Markov models, which have become a computational bottleneck.

**Results:**

We present DeepNOG, an extremely fast and accurate, alignment-free orthology assignment method based on deep convolutional networks. We compare DeepNOG against state-of-the-art alignment-based (HMMER, DIAMOND) and alignment-free methods (DeepFam) on two orthology databases (COG, eggNOG 5). DeepNOG can be scaled to large orthology databases like eggNOG, for which it outperforms DeepFam in terms of precision and recall by large margins. While alignment-based methods still provide the most accurate assignments among the investigated methods, computing time of DeepNOG is an order of magnitude lower on CPUs. Optional GPU usage further increases throughput massively. A command-line tool enables rapid adoption by users.

**Availabilityand implementation:**

Source code and packages are freely available at https://github.com/univieCUBE/deepnog. Install the platform-independent Python program with $pip install deepnog.

**Supplementary information:**

Supplementary data are available at *Bioinformatics* online.

## 1 Introduction

Understanding protein function is a fundamental problem in molecular biology. Characterizing function through biological experiment has very slow throughput, compared to the ongoing growth of sequence data. While functional characterization is time consuming even in model organisms, experiments become particularly complex for currently uncultivatable or otherwise inaccessible organisms, such as intracellular symbionts. The continuous massive stream of sequence data emerging from large-scale genomic and metagenomic projects widens the gap between available raw information and precise data from experiments. Highly efficient and accurate computational methods are, thus, required to extract functional and evolutionary information from all sequence data.

Orthologous relationships are highly informative about protein function (Fitch, 2000; Gabaldón and Koonin, 2013). Phylogenetic and functional information is largely stored in the primary structure of proteins, i.e. the amino acid sequence [Protein sequences contain sufficient information to code for their structure (Possenti *et al.*, 2018). As per the thermodynamic hypothesis, protein sequences determine native conformations (Anfinsen, 1973). It is commonly accepted that protein 3D structures determine function. By transitivity, function (generally) follows sequence.]. Several public resources provide precomputed orthologous groups of protein sequences to aid, for example, comparative genomics and phylogenetics. Among these resources are hand-curated databases, such as COG (Galperin *et al.*, 2015), and (semi-)automatically created databases, such as OMA (Altenhoff *et al.*, 2018) or eggNOG (Huerta-Cepas *et al.*, 2019). The latter is a superset of COG, and additionally comprises over 4 M orthologous groups automatically created from fully sequenced and assembled genomes of 5090 organisms in its latest iteration eggNOG 5. Mapping protein sequences against these well-annotated orthology resources is a popular approach to functional inference, evolutionary classification or metabolic pathway analysis (Galperin *et al.*, 2019).

The problem of grouping protein sequences according to orthologous relationships includes (i) constructing databases of orthologous groups, and (ii) assigning new sequences to precomputed orthologous groups. Here, solely the second subproblem is considered. Several methods for orthology assignments of novel sequences to precomputed groups are available, including alignment-based and alignment-free techniques. Alignment-based methods typically rely on comparisons of multiple sequences, which provide substantially better sensitivity than pairwise sequence alignments. Probabilistic profile hidden Markov models (pHMMs) are used frequently for database search of proteins by sequence homology (El-Gebali *et al.*, 2019). Profile HMMs are derived from multiple sequence alignments (MSAs) and leverage position-specific state transition probabilities for scoring of protein similarity against their corresponding MSA (Eddy, 2011). This fully probabilistic model was found to confer superior performance in detecting homologous protein sequences, particularly for distant relatives, compared to non-probabilistic alignment-based methods like BLAST (Eddy, 2011). For this reason, pHMMs have also seen application specifically in protein orthology mapping (Huerta-Cepas *et al.*, 2017; Mi *et al.*, 2010; Petersen *et al.*, 2017). Hidden Markov models are trained from multiple alignments of protein families or orthologous groups. Inference with all models is then required to assign one particular family or group to a sequence of interest. These steps were rate-limiting factors in previous studies (Feldbauer *et al.*, 2015; Seo *et al.*, 2018). Alignment-based methods are, thus, becoming a computational bottleneck in protein function prediction. This is especially the case for current metagenomic projects. Due to readily available low-cost high-throughput sequencing technologies, millions and soon to be billions of proteins await analysis (see, for example, Pasolli *et al.*, 2019).

Recently, alignment-free deep learning approaches have been applied successfully to numerous biological and biomedical tasks (Eraslan *et al.*, 2019). DeepFam is a deep convolutional network for assigning novel sequences to precomputed orthologous groups (Seo *et al.*, 2018). While it achieves high accuracy and significant speed-up compared to the commonly used pHMM tool HMMER3 (Eddy, 2011), we identify several limitations:

- Sub-optimal scaling to larger datasets.
- COG only (no eggNOG or other large-scale orthology databases).
- Sequence length restrictions.
- Missing user interface for inference (assignments).

To fill these gaps, we introduce the deep network architecture *DeepNOG*. It features superior assignment accuracy and computational efficiency in large orthology databases. DeepNOG allows to assign eggNOG 5 orthologous groups and handles proteins of arbitrary sequence lengths. The Python package deepnog provides researchers with easy-to-use deep learning-based orthologous group assignment. Note, that DeepNOG’s scope does not encompass the delineation of new groups, or further disentanglement of protein relationships within individual groups.

## 2 Materials and methods

In this section, we introduce the orthology databases, deep network architectures and a metagenomic dataset used for DeepNOG evaluation.

### 2.1 Orthology databases

We used two orthology databases for our experiments: Our primary interest is eggNOG, a large public database with semi-supervised, fine-grained orthologous groups on multiple levels of the tree of life. We demonstrate fast and reliable orthologous group assignments in eggNOG 5 in Section 4.3. COG is a comparatively small, manually curated database by NCBI that serves here for comparison to previous results of competing methods.

Table 1 gives an overview of the most important characteristics and differences between COG (2014) and eggNOG 5. For the latter, we considered sequences with exactly one orthologous group (OG) assignment at the bacteria level. The population of orthologous groups is typically highly skewed. While the expected population size of each OG in the top taxonomic levels is roughly in the order of one hundred members, there exist many OGs comprising few members, or even a single sequence (singletons). Conversely, relatively few groups with high cardinality contain a large fraction of all sequences in the database. In the machine learning context, datasets highly imbalanced with respect to class cardinalities pose a challenge to both traditional and modern methods (Johnson and Khoshgoftaar, 2019), some consequences of which are being discussed in the results Section 4.1. When technically necessary, we disregarded OGs below a certain member threshold. This typically only affected a relatively low number of sequences. Additional details on database characteristics are provided in Supplementary Section SC.1.

**Table 1.** Main characteristics of orthologous groups databases

	COG 2014	eggNOG 5 (bacteria)
Number of proteins	1 674 176	13 836 642
Number of OGs	4631	206 782
OG population range	1–10 632	1–97 670
Sequence length range	21–29 202	23–24 921

#### 2.1.1 COG: Clusters of Orthologous Genes

The Clusters of Orthologous Genes database was introduced in 1997 to provide evolutionary classification of protein families. COG is a relatively small, but manually curated and, thus, high-quality orthology resource. Orthologous groups are constructed by considering bidirectional best hits of sequences comparing complete genomes.

COG was used in Seo *et al.* (2018) for evaluating DeepFam in terms of accuracy and speed in comparison to HMMER. Here, we used COG to reproduce those results, and as a baseline to compare the new DeepNOG architecture with its competitor DeepFam and alignment-based methods. For a broader comparison of alignment-free methods including k-mer-based algorithms the reader is referred to Seo *et al.* (2018). Since identical datasets were used for benchmarking, and DeepFam had been found to outperform the competing methods, those comparisons were not re-iterated here, but only the state-of-the-art alignment-free method was considered.

#### 2.1.2 EggNOG 5

The *evolutionary genealogy of genes: non-supervised orthologous groups* database (eggNOG) builds upon the COG database. In addition to this supervised part, a large fraction of the database is constructed by an unsupervised clustering algorithm (Huerta-Cepas *et al.*, 2019). The resulting orthologous groups are often referred to as NOGs or ENOGs. In its current version 5, eggNOG consists of 4.4 million orthologous groups distributed across 379 taxonomic levels. For this work, we primarily considered single-label proteins, following the approach of DeepFam (Single-label proteins are associated with only one OG, which excludes, for example, certain multi-domain proteins. For effect sizes see Supplementary Table S1.). We focused on the bacterial level, which is highly relevant for metagenomic studies. For example, reliable phenotypic trait prediction from complete or incomplete genomes has so far been demonstrated for bacteria only (Feldbauer *et al.*, 2015; Weimann *et al.*, 2016), and is used, for instance, in the PhenDB pipeline (PhenDB: https://phendb.csb.univie.ac.at/). The methodology is, however, not limited to specific taxonomic levels. DeepNOG is also evaluated on the eggNOG 5 root level.

#### 2.1.3 Human infant gut metagenomes

The human gut microbiome has received extensive attention and has been linked to numerous medical conditions, including infectious diseases, obesity or cancer (Cani, 2018). Three human infant gut metagenome studies (SRP069019, SRP090628 and SRP056054) were selected exemplarily for orthologous group annotation with particular focus on the type VI secretion system (T6SS). From each infant, the sample with the largest number of high- and medium-quality metagenome-assembled genomes (MAGs) was selected to form a set of 3337 MAGs.

### 2.2 Convolutional networks for OG assignment

Both the newly developed DeepNOG architecture and DeepFam rely on convolutional units to extract informative subsequence patterns from proteins for orthologous group assignment. That is, both are supervised *end-to-end* learning methods, which do not require manual feature extraction, such as k-mer frequencies. Instead, they take raw protein sequences as input and return class labels for each sequence. In this section, we describe common features and important differences between these methods.

#### 2.2.1 Architectural comparison of DeepNOG and DeepFam

**Fig. 1.**
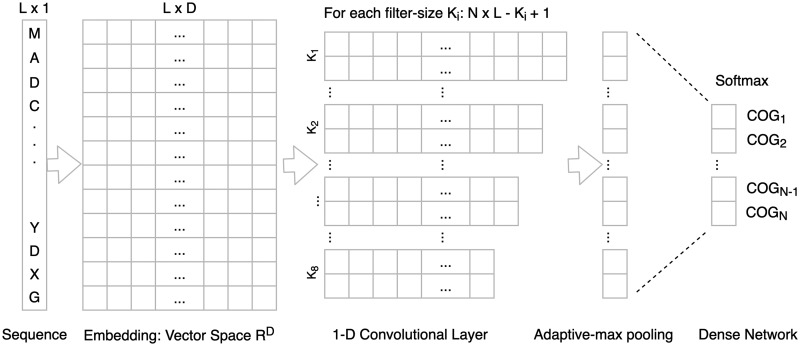
DeepNOG network architecture

DeepNOG is a convolutional network architecture inspired by DeepFam with multiple improvements to overcome its limitations including the restriction to fixed length protein sequences, and applicability to eggNOG. Figure 1 provides a visual overview of the DeepNOG architecture (see Supplementary Fig. S3 for the DeepFam architecture). The following subsections give a brief overview of DeepFam and introduce the architectural improvements incorporated into DeepNOG. The influence of each of the introduced architectural changes compared to DeepFam was investigated in an ablation study (see Supplementary Section SD.1).

#### 2.2.2 Encoding layer: word embeddings

Proteins are commonly represented in human-readable form, such as the FASTA format, which contains the sequence encoded in the IUPAC one-letter amino acid code. For deep learning, sequences must be transformed into a format processible by deep networks, typically numerical vectors. Therefore, categorical data are frequently embedded into vector spaces by one-hot encoding: Vector length is determined by the alphabet size (for example, the number of proteinogenic amino acids), and the vector for a particular amino acid is 1 in a cell specific for this amino acid, and 0 everywhere else. One-hot encodings hence exhibit equidistance between all categories. Clearly, this is an undesirable feature for amino acids, which naturally cluster with respect to chemical and biological features or size.

DeepFam employs a pseudo one-hot encoding: The 20 standard amino acids and the ‘X’ character (unknown) are one-hot encoded, yielding a 21-dimensional embedding. Three ambiguous codes are allowed to interpolate between their manifestations (e.g. J = $\frac{1}{2}$I + $\frac{1}{2}$L). In sequence alignments, substitution matrices such as BLOSUM, PAM or PSSMs are employed to factor in similarities between individual amino acids. DeepNOG accounts for these similarities by embedding each of 26 amino acid codes (using Biopython’s extended IUPAC protein alphabet) into ${\mathbb{R}}^{D}$ (‘word embedding’). These ***D*-dimensional vector representations** are jointly trained with the network. That is, the embedding layer has flexibility to learn common properties of amino acids by placing them in ${\mathbb{R}}^{D}$ so that distances in this space reflect biochemical (dis-)similarities. The word embedding dimension *D* can be lower than the alphabet size, and thus, lower than the one-hot encoding dimension. For DeepNOG, *D *=* *10 gave best results on validation data, which reflects the findings of a recent survey on amino acid encoding schemes (ElAbd *et al.*, 2020). Consequently, each amino acid is represented as a ten-dimensional vector. Concrete realizations were obtained for each model individually during training. Conveniently, the learned amino acid embeddings can be visually inspected for biological plausibility, which enhances the interpretability of trained models (see Fig. 3). The embedded sequences are passed to feature extraction in the convolutional layer.

#### 2.2.3 Convolutional layer

After the embedding, a 1-D convolutional layer is employed in DeepFam. The *i*th row in the convolutional layer corresponds to a filter of size K_*i*_ which is applied to L-K_*i*_+1 consecutive (encoded) protein subsequences of length K_*i*_ resulting in L-K_*i*_+1 convolutional units (with L, the protein sequence length). 1-D means that the filter treats each column in the encoding as a separate input channel. If the amino acid alphabet consists of C letters, this results in the filter learning C vectors of weights (tunable network parameters), each of length K_*i*_. Therefore, the *k*th element in a weight vector corresponds to the weight given to a certain amino acid occurring in the *k*th position of the looked upon protein subsequence. The 1-D convolutional operation is followed by an activation function (non-linearity). DeepFam uses rectified linear units (ReLU) as activation functions. Furthermore, it chooses eight different filter sizes (8, 12, 16, 20, 24, 28, 32 and 36). In the most successful parametrization, it uses 250 different filters for each filter-size resulting in 2000 independent filters. Seo *et al.* (2018) showed that the learned filters are sensitive to sequence motifs typical for certain orthologous groups, and their occurrences can distinguish orthologous groups, underlining successful feature extraction by the convolutional layer.

DeepNOG’s convolutional layer closely resembles the architecture of DeepFam but exchanges the ReLU activation function for scaled exponential linear units (SELU) to obtain a **self-normalizing network**: Activations converge toward zero mean and unit variance, without an additional explicit normalization step, such as batch normalization. This eliminates the vanishing or exploding gradients problems, enables strong regularization and yields stable learning (Klambauer *et al.*, 2017; see also Section 2.2.6).

In DeepNOG, employing a 1-D convolution means that each dimension in the embedding vector space ${\mathbb{R}}^{D}$ is treated by the filter as an independent input feature vector. The filter then weights the importance of the positive or negative strength of a feature at a certain position in the input protein subsequence for each input feature vector separately. The positive or negative strength of a feature at a certain position is determined by the concrete amino acid in this specific position in the subsequence. Thus, the interpretation of filters getting sensitive to discriminatory motifs for OG assignments, as put forth by Seo *et al.* (2018), holds.

#### 2.2.4 Pooling layer

DeepFam employs a standard 1-max-pooling layer. This means, the *i*th node in the pooling layer corresponds to the maximum value in the *i*th row of the convolutional layer and all other values are discarded. 1-max-pooling layers require the length of the input rows to be specified exactly. For this reason, DeepFam fixed the length L of input sequences to 1000, zero-padded smaller input sequences and discarded longer ones. Both COG and eggNOG, however, comprise sequences of very high length variability (see Table 1, and Supplementary Fig. S1). Therefore, this is a significant limitation for the applicability of this architecture to arbitrary sequences in COG and eggNOG as well as for classifying arbitrary user sequences.

To this end, DeepNOG exchanges the pooling layer for an adaptive max-pooling layer. It also extracts the maximum value in an input row of the convolutional layer but can handle arbitrary input sizes and has, therefore, no upper bound on the length of the input sequences. Extremely short input sequences of lengths smaller than the biggest filter size K are zero-padded to length K for training and inference (where *max*(*K*) = 36 in DeepNOG). DeepNOG has, consequently, **no restriction on protein sequence length**.

#### 2.2.5 Classification layer

DeepFam aggregates activations from the feature extraction subnetwork in a fully connected subnetwork with one hidden layer followed by a softmax for classification. In the most successful parametrization of DeepFam, it uses 2000 units in the hidden layer. The output at each node can be interpreted as the confidence the network has in associating the input protein sequence with the specific orthologous group corresponding to the node (Goodfellow *et al.*, 2016). The OG assignment is finally based on the highest confidence among the output nodes.

DeepNOG directly places the softmax output layer after the adaptive max-pooling layer, thereby omitting the additional hidden layer. This **reduces the total number of parameters** and improves run time [The number of model parameters is not necessarily a good measure of its complexity, and deep networks have been shown to generalize better in overparametrized regimes (Belkin *et al.*, 2018). Here, reducing the number of parameters should be seen in the light of increasing training and inference speed rather than as a regularization method.]. Since softmax layers are equivalent to logistic regression, DeepNOG can be understood as a two-stage machine learning model. In the first stage, it extracts the (strength of) occurrence of sequence motifs with discriminatory information regarding OGs. In the second stage, it applies logistic regression, using the (strength of) occurrence of sequence motifs as input, to calculate probabilities of all orthologous groups in the model. Finally, the input sequence is assigned to the OG with the highest probability.

#### 2.2.6 Hyperparameters and training procedure

DeepNOG is trained as a self-normalizing network with scaled exponential linear units (SELU) as activation functions. DeepNOG with SELUs achieved better empirical results (on validation data) than with ReLU plus batch-normalization approach taken by DeepFam, while at the same time being computationally more efficient. The convolutional and dense layers of DeepNOG are initialized as described in Klambauer *et al.* (2017). Dropout with *P *=* *0.3 is employed for regularization. Additionally, alpha-dropout with P={0.05,0.10,0.20,0.30} as suggested in Klambauer *et al.* (2017) as well as dropout with *P *=* *0.5 as suggested by Hinton *et al.* (2012) were investigated but found to perform inferior (on validation data). Due to computational limitations and the similarity of COG to eggNOG, the performance of hyperparameter choices on COG was used as an estimator for the performance on eggNOG. Therefore, DeepNOG uses identical parametrization and the same filter sizes 8, 12, 16, 20, 24, 28, 32 and 36 as DeepFam and 150 filters for each size. For stochastic optimization, Adam (Kingma and Ba, 2014) was used with a learning rate set to 0.01 in conjunction with a scheduler decreasing the learning rate by 25% after each epoch. Additional Adam parameters were set to default values. Batch size was set to 64, unless GPU memory was exceeded, in which case the batch size was reduced to fit into memory. Training was performed on an Nvidia P6000 GPU with 24 G memory. For some experiments, the memory requirements of the best DeepFam parametrization exceeded the total memory on the available hardware. Therefore, an additional parametrization was trained, which used 150 instead of 250 convolutional filters for each filter size, and 1500 instead of 2000 hidden units in the classification layer. These design decisions are in accordance with investigations of different hyperparameter choices in Seo *et al.* (2018), which reported only a minor increase in error (<1%-point) for the more lightweight parametrization. We refer to these models as ‘DeepFam light’.

### 2.3 Alignment-based orthologous group assignment

DeepNOG is also compared with alignment-based methods achieving state-of-the-art performance in orthologous group assignment, which are used in eggNOG-mapper (Huerta-Cepas *et al.*, 2017), the official OG-assignment tool by the eggNOG consortium. Comparison is against the core methods rather than eggNOG-mapper, because the tool performs additional steps, such as gene ontology assignments. The complete eggNOG-mapper pipeline would, therefore, be at a disadvantage in timing experiments.

#### 2.3.1 Profile hidden Markov models

Profile hidden Markov models (pHMMs) drive eggNOG-mapper v1, and can optionally be chosen in v2.

For experiments on COG and eggNOG, multiple sequence alignments were built for each orthologous group with FAMSA v1.3.2 (Deorowicz *et al.*, 2016) restricted to training set proteins. Profile HMMs were created from these alignments with hmmbuild (HMMER 3.3). Test set inference was performed by hmmsearch. Default parameter values were used, unless explicitly specified otherwise. Each query was assigned to the OG with the lowest corresponding inference e-value. Inference speed was measured with hmmsearch with parameters –noali –cpus 1. Parallel queries were handled by the hmmpgmd daemon.

#### 2.3.2 DIAMOND

DIAMOND is the main algorithm driving eggNOG-mapper v2. DIAMOND databases were created for each training set with diamond makedb. Test set inference was performed by diamond blastp with parameters –more-sensitive -e 0.001 –top 3 –query-cover 0 –subject-cover 0 to mimic eggNOG-mapper.

### 2.4 Training, validation, test splits

Unbiased estimation of the true performance of machine learning models requires splitting data into independent training, validation and test sets, or nested cross-validation. We employed different split strategies for the experiments described below.

Experiments on COG used a single cross-validation scheme. Training and validation (model selection) are performed on a common subset, while performance estimation uses an independent test set, which replicates the procedure outlined in Seo *et al.* (2018). This possibly resulted in too pessimistic assignment performance estimates due to selecting models of suboptimal generalization power. However, this procedure is required for replicating DeepFam experiments and a fair comparison to DeepNOG.

For eggNOG experiments, we refrained from cross-validation given the large database size. Models were trained and selected on training/validation splits. Assignment performance was estimated on an independent test set. All splits were stratified according to class labels, with a split ratio of 81%, 9% and 10% of all sequences, for training, validation and test sets, respectively.

This procedure might report optimistic error rates for biological sequences, if highly similar sequences are present in both training or validation and test set. For this reason, we trained and evaluated additional models on eggNOG 5 sequences (root and bacteria levels) clustered according to UniRef50 and UniRef90 (release 2019_11, Suzek *et al.*, 2015) to estimate possible biases of this type.

## 3 Implementation

The Python tool deepnog provides fast protein orthology assignments with PyTorch deep networks (Paszke *et al.*, 2019). At the time of writing, it supports the DeepNOG architecture as described in Section 2.2 trained on the root (tax 1) and bacteria (tax 2) levels of the eggNOG 5 database out of the box. The tool is agnostic toward specific network architectures and orthology databases. Additional networks for arbitrary orthology databases can be registered easily. This requires only the PyTorch model definition, and the corresponding trained weights. The deepnog train command allows users to train custom models for additional taxonomic levels of eggNOG, or even different databases, such as OMA. For deepnog usage details the reader is referred to the user & developer guide available online (User & developer guide: https://deepnog.rtfd.io). deepnog can be installed from the Python Package Inventory by $pip install deepnog on all major operating systems. Source code, issue tracker and additional links are available on https://github.com/univieCUBE/deepnog. Further information about software development principles, tests, continuous integration or documentation is available in Supplementary Section SA. Refer to Supplementary Section SB for notes on data availability.

DeepFam was originally implemented using the TensorFlow framework. For fair comparison, we re-implemented the network in PyTorch, and reproduce the findings reported by Seo *et al.* (2018) for the COG database in Section 4.2, where we also compare DeepNOG with alignment-based methods.

## 4 Results

DeepNOG was first evaluated on both COG and eggNOG 5, and compared with DeepFam, HMMER and DIAMOND.

We then proceeded to the larger eggNOG 5 database, for which we report fast and accurate assignments with DeepNOG in Section 4.3. For an application example, we consider the search for type VI secretion systems in metagenomic data in Section 4.4. Additional experiments on related classification tasks (protein fold, and GPCR family assignments) are presented in Supplementary Sections SD.3 and SD.4, respectively.

### 4.1 Addressing imbalanced orthologous group populations

Both COG and eggNOG 5 constitute highly imbalanced datasets with respect to group membership. Learning to correctly assign orthologous groups with population sizes below a certain threshold is challenging. For example, in the extreme case of singletons, it is impossible to perform train-test splits. Generalization performance cannot be estimated in such cases. Therefore, Seo *et al.* (2018) introduced three minimum population thresholds for OGs (100, 250 and 500) and investigated, how the method scales with the number of OGs and level of imbalance. We consider the minimum population thresholds of 100 and 500 for comparison between DeepNOG and DeepFam.

Imbalanced datasets also require a careful choice of performance measures. For direct comparison with previous results, we report classification accuracy, which is the fraction of correct assignments in all assignments. This measure is biased toward the performance of the largest groups. In order to investigate assignment performance for orthologous groups with few members, we also report macro-averaged precision and recall. That is, precision and recall are computed for each group and averaged over all groups. Each orthologous group, therefore, contributes equally to the overall performance. Accuracy higher than both macro-averaged measures suggests that the model performs better on large groups than on small groups.

### 4.2 DeepNOG versus state-of-the-art methods on COG

DeepFam processes sequences to a maximum length of 1000. Applying the length threshold to COG removes 1.3% of the protein sequences (see Supplementary Table S2). Additionally requiring a minimum population of 100 removes 6.5% of sequences.

**Fig. 2.**
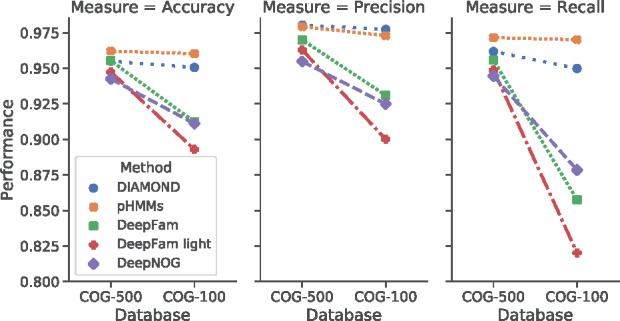
Assignment accuracy for COG (minimum member threshold 500 and 100)

Figure 2 reports classification performance of DeepNOG and competing assignment methods on two COG subsets. The results were obtained by averaging three-fold cross-validation results. Datasets and splits are identical to those used by Seo *et al.* (2018) to allow for a fair comparison. Profile hidden Markov models (pHMMs) generated with HMMER, and DIAMOND provided the baseline for alignment-based orthologous group assignment. We investigated a lightweight version (‘DeepFam light’) in addition to the best parametrization of DeepFam, which was not trainable on the available resources for eggNOG (see Section 2.2.6).

The alignment-based methods achieved best assignment quality, with pHMMs profiting from high recall (sensitivity). Accuracy of DeepFam was in very good agreement with previously published values (Seo *et al.*, 2018). Note that we obtained 4%-pts higher accuracy for pHMMs compared to the values provided in the same source. The alignment-free methods also yielded highly accurate assignments in COG-500 but showed reduced performance in the larger COG-100 dataset. DeepNOG scaled better to larger datasets than both DeepFam variants. Section 4.3 shows that this effect is amplified in the larger eggNOG database.

Inference speed is an important constraint for applications of orthologous group assignment. Again, the deep learning based methods were compared with alignment-based methods. For fair comparison, all timings were performed on the same machine on a single CPU core [1000 proteins, AMD Opteron(tm) Processor 6320 @ 1.80 GHz]. DeepNOG was slightly faster than DeepFam, and **up to an order of magnitude faster** than the alignment-based methods (see Table 2).

**Table 2.** Inference time (seconds/1000 sequences) for COG and eggNOG 5 (bacteria level)

	COG-500	COG-100	${\text{NOG}}_{2}^{5}$-500	${\text{NOG}}_{2}^{5}$-100
DIAMOND	161.7	214.5	781.6	810.0
pHMMs	96.3	207.0	218.9	253.7
DeepFam	49.0	50.2	n/a	n/a
DeepFam light	32.7	35.0	34.9	38.7
DeepNOG (CPU)	**24.3**	**26.0**	**26.4**	**28.9**
pHMMs (parallel)	4.8	5.1	9.5	14.4
DeepNOG (GPU)	0.6	0.6	0.6	0.6

*Note*:Fastest method **bold** (single core). Averages over three replicates. Parallel pHMMs used 29x16 CPU cores.

We conclude that DeepNOG (i) provides high assignment performance, (ii) scales better to higher numbers of groups and more imbalanced datasets and (iii) is faster than its alignment-free competitor on COG.

### 4.3 Evaluation of eggNOG assignment

The much larger eggNOG 5 database is our primary interest. Similar to our experiments on COG, we applied the minimum orthologous group population threshold 100 to eggNOG, yielding datasets ${\text{NOG}}_{1}^{5}$-100 and ${\text{NOG}}_{2}^{5}$-100, where superscripts and subscripts indicate eggNOG version and taxonomic level, respectively (1, root; 2, bacteria). Since DeepNOG was designed to handle sequences of arbitrary length, we did not apply any sequence length restrictions to eggNOG. See Supplementary Table S2 for details on the resulting datasets used for the experiments in this section.

#### 4.3.1 Amino acid embedding

**Fig. 3.**
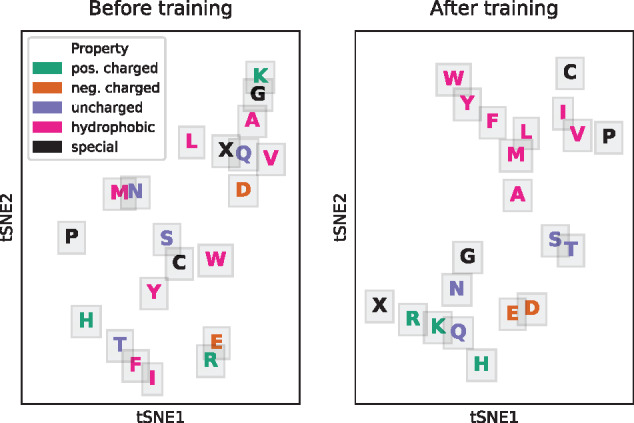
Amino acid representations in the encoding layer of DeepNOG. Random initialization before training (left), and tuned representations after DeepNOG training on ${\text{NOG}}_{2}^{5}$-100 (right). Amino acids are colored based on biochemical properties. Ambiguous codes that were not present in the dataset are excluded from the plots

The word embedding layer used for encoding the input amino acids is one important feature of the DeepNOG architecture. Figure 3 visualizes the amino acid embeddings of the DeepNOG eggNOG 5 (bacteria) model before and after training. Amino acids were initially positioned randomly in ${\mathbb{R}}^{10}$. Consequently, no meaningful cluster structures were visible before training. After training, the learned embeddings exhibited biologically plausible clusters matching well with biochemical properties. Furthermore, a finer topology could be identified. For example, the aromatic amino acids Phenylalanine (F), Tryptophan (W) and Tyrosine (Y) were distinctly grouped together. In addition, amino acids with unique features, such as disulphide-bond forming Cytosine (C), or the structural disruptor Prolin (P), were located on cluster borders (Note that inter-cluster distances typically hold no meaning in t-SNE visualizations, which optimize distance distributions between neighbors only.). The embeddings in ${\mathbb{R}}^{10}$ were reduced to ${\mathbb{R}}^{2}$ by t-SNE (Maaten and Hinton, 2008).

#### 4.3.2 Assignment quality

**Fig. 4.**
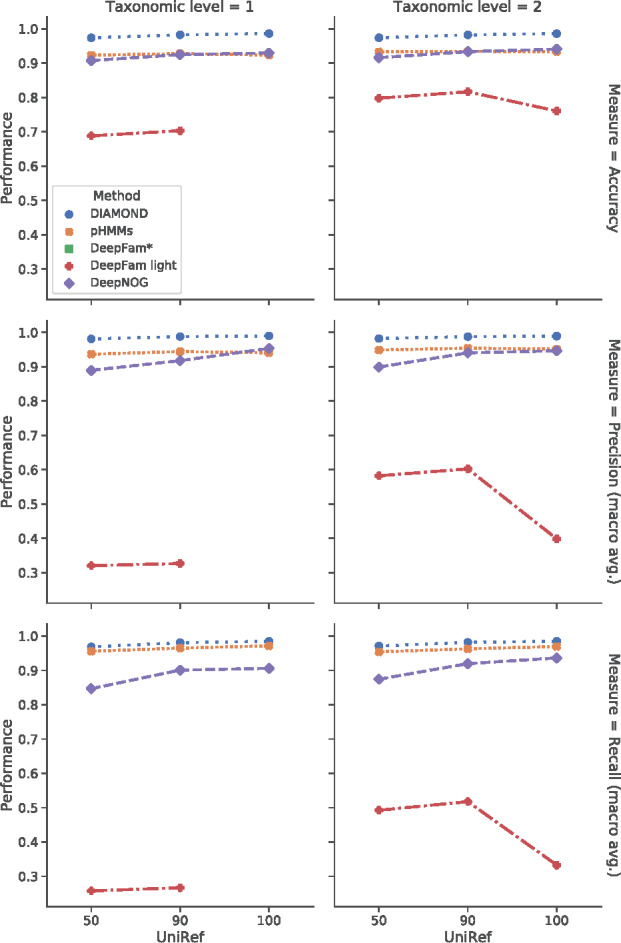
Assignment accuracy for ${\text{NOG}}_{1}^{5}$-100 (tax 1) and ${\text{NOG}}_{2}^{5}$-100 (tax 2) and three clustering regimes. Note: * DeepFam not trainable on 24 G GPU for eggNOG datasets

Figure 4 reports assignment performance of DeepNOG for eggNOG 5 datasets. In order to control possible biases stemming from similar sequences in training and test sets, we performed experiments on different datasets with sequences clustered to 50%, 90% and 100% identity (that is, UniRef50, UniRef90 and UniRef100, respectively). Supplementary Table S3 details the resulting datasets. We compared our method with DeepFam light, since these eggNOG 5 datasets contain more classes (orthologous groups) than COG, so that the best DeepFam parametrization exceeded the available memory (see Section 2.2.6).

DeepNOG showed very high assignment accuracy on the eggNOG 5 root and bacteria datasets on all three clustering levels. While the number of orthologous groups was more than doubled (bacteria) and quadrupled (root) compared to COG, performance measures were nearly stable, with accuracy slightly below COG results. We observed only minute differences between accuracy, precision and recall on the unclustered (UniRef100) datasets, clearly demonstrating DeepNOG’s excellent scaling to larger numbers of classes and population imbalance. Stricter clustering impaired macro averaged recall, indicating more difficult assignments to small clusters in remote homology regimes. DeepNOG was slightly more precise than sensitive, similar to other alignment-free methods.

DeepNOG outperformed DeepFam light with at least ten percent-points difference in accuracy, and striking differences in macro averaged precision and recall on ${\text{NOG}}_{2}^{5}$-100. This indicates substantial difficulties to predict rare orthologous groups correctly with DeepFam. The performance drop in ${\text{NOG}}_{2}^{5}$-100-UniRef100 and ${\text{NOG}}_{1}^{5}$-100-UniRef50, 90and 100 was in agreement with DeepFam’s suboptimal scaling to larger datasets.

Profile hidden Markov model performance was on par with DeepNOG. They were less affected by sequence similarity effects (UniRef clustering), and provided higher sensitivity. DIAMOND again achieveed the highest overall assignment quality. As to be expected, both alignment-based methods excelled in terms of accuracy. However, this came at high computational cost, as discussed below.

#### 4.3.3 Assignment speed

Table 2 provides execution times on the eggNOG 5 bacteria datasets. The experiment setup was identical to the setup for COG (see Section 4.2). The timings for both alignment-based methods were in agreement with the initial hypothesis, that they are becoming a computational bottleneck. Inference time using profile HMMs or DIAMOND scaled unfavorably to larger databases. While pHMMs scaled approximately linearly with the number of classes, DIAMOND scaled proportionally to the number of sequences in the database (see Supplementary Section SD.2 for details). The deep learning-based alignment-free methods were faster overall, and scaled decisively better to many classes and sequences. Note, that deepnog supports GPUs, which increase throughput significantly. DeepNOG (GPU) outperformed a massively parallel setup of HMMER using the hmmpgmd daemon (see Table 2, lower part) (Note that suboptimal scaling to large numbers of parallel threads is a known limitation of HMMER3.3, see http://eddylab.org/software/hmmer/Userguide.pdf (Introduction, p. 14).). At the same time, DeepNOG models had a smaller memory footprint than alignment-based methods. The network weights for ${\text{NOG}}_{1}^{5}$-100 and ${\text{NOG}}_{2}^{5}$-100 amounted to 72.4 M and 31.5 M, respectively. The corresponding pHMMs required 7.2 G and 1.9 G, and DIAMOND databases used 6.5 G and 3.9 G of memory.

In summary, DeepNOG scaled well from smaller databases like COG to larger databases like eggNOG. DeepNOG showed powerful performance on eggNOG 5 in terms of assignment accuracy, execution speed and memory footprint. It clearly outperformed DeepFam in all of the evaluated metrics, and provided accuracy on par with pHMMs at a fraction of their computational cost.

#### 4.3.4 Assignment confidence threshold

Classifiers typically *partition* data: Every object is assigned to one of the known classes. This is not always desirable, especially, when there are additional classes that are not part of a model. Any objects from these classes would be labeled incorrectly. For example, human protein sequences fed into a bacteria-level model would be assigned to inappropriate orthologous groups. Instead, one might not want to assign any OG labels in these cases.

**Fig. 5.**
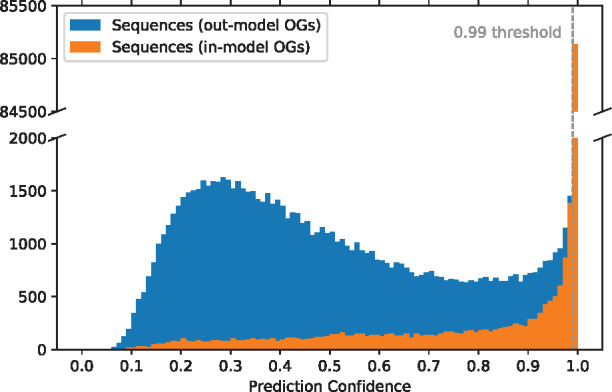
Assignment confidences (bin size 0.01)

Deep networks with a softmax classification layer provide output that represents a probability distribution over the available classes. The output at a single neuron can, therefore, be interpreted as the network’s confidence, that the input belongs to the corresponding class among the available classes. DeepNOG supports setting an assignment confidence threshold to decide, whether an input protein can be associated with any OG included in the model. We investigated the threshold empirically on the ${\text{NOG}}_{2}^{5}$-100 dataset by comparing the probability distribution of sequences from *in-model* OGs to the distribution of sequences from *out-model* OGs. 100 000 proteins were randomly sampled from the ${\text{NOG}}_{2}^{5}$-100 test set. These were members of orthologous groups included in the model but had not been seen during training. Furthermore, 100 000 proteins were randomly sampled from orthologous groups in eggNOG 5 (bacteria) that were not included in ${\text{NOG}}_{2}^{5}$-100. Their true corresponding OGs were not included in the model. All proteins were classified with DeepNOG and the probabilities of the assigned groups were investigated. Figure 5 depicts the overlayed histograms. In-model sequences showed a distinct distribution, well separable from the distribution of out-model sequences. That is, the network was typically highly confident for proteins of known OGs, while in most cases it was much less confident about proteins from OGs unknown to the model.

A sensible confidence threshold can be set to a value on the Pareto boundary of minimizing false positives and false negatives, depending on the specific requirements of downstream experiments. We set the default threshold to 99%. In Figure 5, there are 1450 out-model sequences and 1382 in-model sequences at this level. Bins below are clearly dominated by unknown OGs, while the bin above contains a vast majority of all in-model sequences. This is a rather strict threshold avoiding false positives. deepnog allows to set this threshold individually per experiment.

### 4.4 T6SS in metagenomic data

**Fig. 6.**
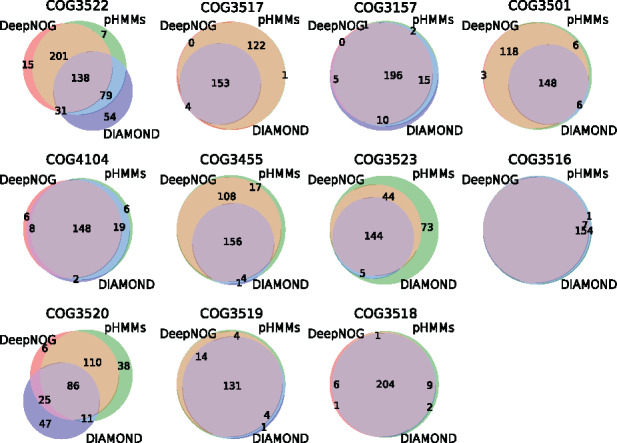
T6SS assignment overlap of different methods

The collection of metagenome-assembled genomes (MAGs) from human infant gut samples was annotated with DeepNOG, eggNOG-mapper (version 2.0.0, using the DIAMOND backend) and HMMER (pHMMs). Figure 6 shows the assignment overlap of the three different methods for eleven T6SS-related COGs (Ho *et al.*, 2014). Supplementary Table S7 maps COG identifiers to the respective T6SS components. Assignment patterns were nearly identical for all methods in five COGs. In three COGs, DeepNOG and HMMER yielded highly similar annotations, while eggNOG-mapper/DIAMOND detected fewer T6SS components. There was more disagreement on the remaining COGs, but DeepNOG assignments were always highly similar to assignments by HMMER. Overall, DeepNOG retrieved T6SS components from MAGs yielding large overlap with alignment-based methods, indicating the methods’ applicability to real metagenomic data.

## 5 Discussion and outlook

We introduce DeepNOG, a deep convolutional network for protein orthologous group assignment. It was shown to achieve high accuracy for both the COG and eggNOG databases, superior to the state-of-the-art alignment-free method DeepFam. Highest assignment accuracy is still achieved by alignment-based tools (DIAMOND, HMMER), which predominantly stems from their higher sensitivity (recall) compared to alignment-free methods. DeepNOG is computationally more efficient than alignment-based methods, providing higher throughput of protein sequences on CPUs and particularly on GPUs. We believe that this feature will help researchers to keep up with the never-ending stream of newly sequenced genomes and metagenomes. In addition, we showed that the network extracts biologically meaningful information from sequences, and automatically clusters amino acids in a way that matches biochemical properties. The Python program deepnog allows users to effortlessly apply the introduced methods, and classify their sequences for eggNOG 5 groups.

Some limitations of DeepNOG remain to be tackled in future work. We currently provide models only for eggNOG 5 root and bacteria levels. Training models for additional levels is in principle straight-forward but requires non-negligible compute resources. We will provide additional models on demand. As an alternative, deepnog enables users to train further models themselves. Second, orthologous groups with very few members still pose a challenge for deep networks, as the number of parameters increases with the number of groups, and few examples are available per class. To this end, we will investigate softmax approximations, such as, for example, presented by Grave et al. (2017). At the moment, deepnog may be used in combination with another tool, such as eggNOG-mapper. DeepNOG typically assigns a large fraction of sequences. By applying an assignment confidence threshold, users can feed the remaining unassigned sequences to the other tool at the cost of slightly increased computation time for full coverage of rare orthologous groups. Third, DeepNOG is currently limited to single-label classification, that is, it cannot assign several orthologous groups to a single sequence. DeepNOG can be extended to the multi-label setting by applying few changes to the architecture and training procedure. However, highly imbalanced datasets typically pose a challenge to multi-label classification. Future experiments will show, whether extended DeepNOG networks can provide reliable multi-label orthologous group assignments.

Finally, we follow recent advances in bioinformatics based on deep learning with great interest, especially the advent of transfer learning. It is widely believed, that transfer learning played a major role in machine learning breakthroughs in computer vision with convolutional networks pretrained on ImageNet (Deng *et al.*, 2009), or in natural language processing with recurrent networks or transformer networks pretrained on large text corpora (e.g. Devlin *et al.*, 2018; Lan *et al.*, 2019). Recently, Rives *et al.* (2019) and Strodthoff *et al.* (2020) reported on unsupervised pretraining of protein sequence representations. While preliminary experiments with UDSMProt finetuned for OG assignment did not yet yield improved results, we believe this direction of research to be highly promising.

Furthermore, the related but distinct problem of constructing orthologous groups could be tackled by modern unsupervised or reinforcement deep learning approaches. Orthology databases have long applied unsupervised learning, such as clustering (Li, 2003). Clustering proteins via sequence vector representations would allow for alignment-free orthology construction, using representations as described above, or learned in alternative schemes, such as twin or triplet deep networks (Chen *et al.*, 2020; Zheng *et al.*, 2019). A plethora of general and deep learning-based clustering methods are available (Aljalbout *et al.*, 2018; Karim *et al.*, 2020). Reinforcement learning has attained less but increasing attention in the field (Mahmud *et al.*, 2018). It will be interesting to see, whether playing the ‘game’ of sequence evolution can help in deriving orthologous relationships.

## Supplementary Material

btaa1051_Supplementary_Data
