# Supplementary figures and images for: DeepNOG: fast and accurate protein orthologous group assignment
Source: Bioinformatics. 2020 Dec 26;36(22-23):5304–12. doi: 10.1093/bioinformatics/btaa1051 (PMC8016488; doi:10.1093/bioinformatics/btaa1051)

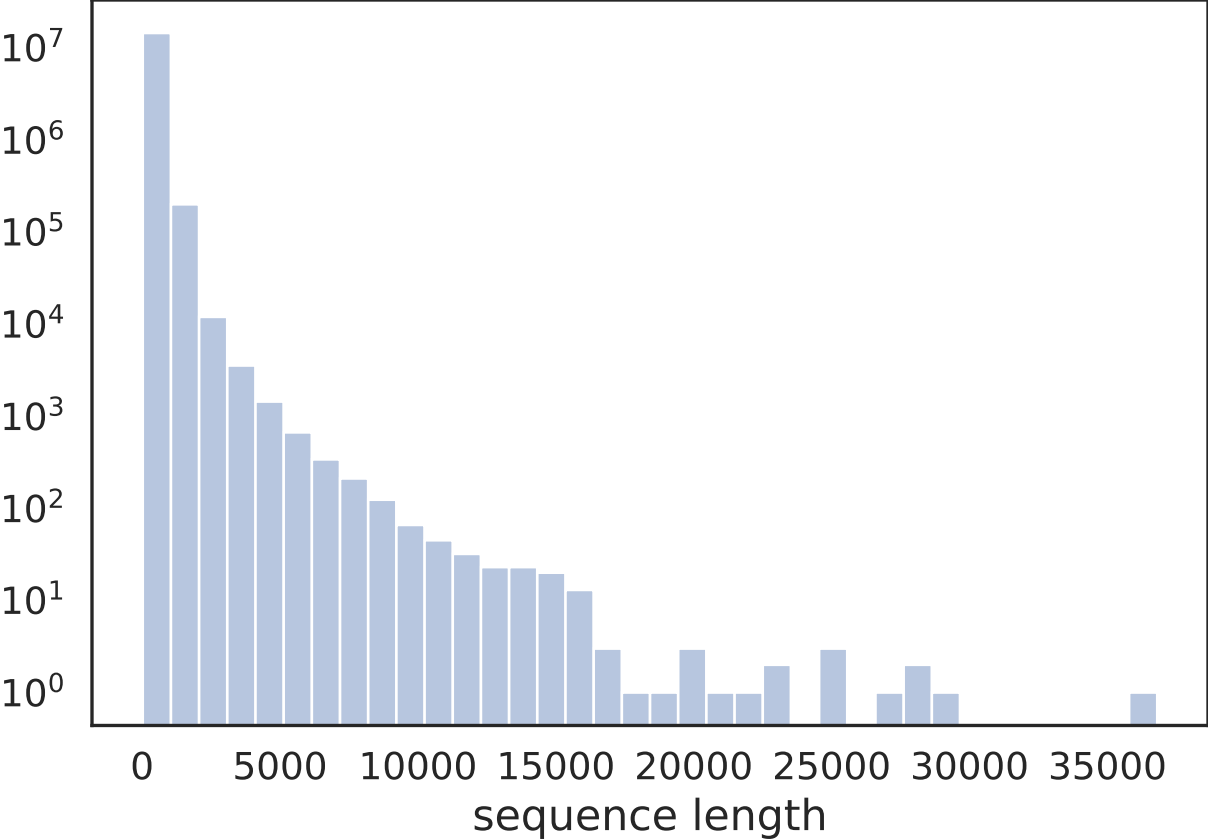

Supplement: btaa1051_Supplementary_Data [file btaa1051_supplementary_data.zip › sequence_lengths_eggNOG5_2.pdf]

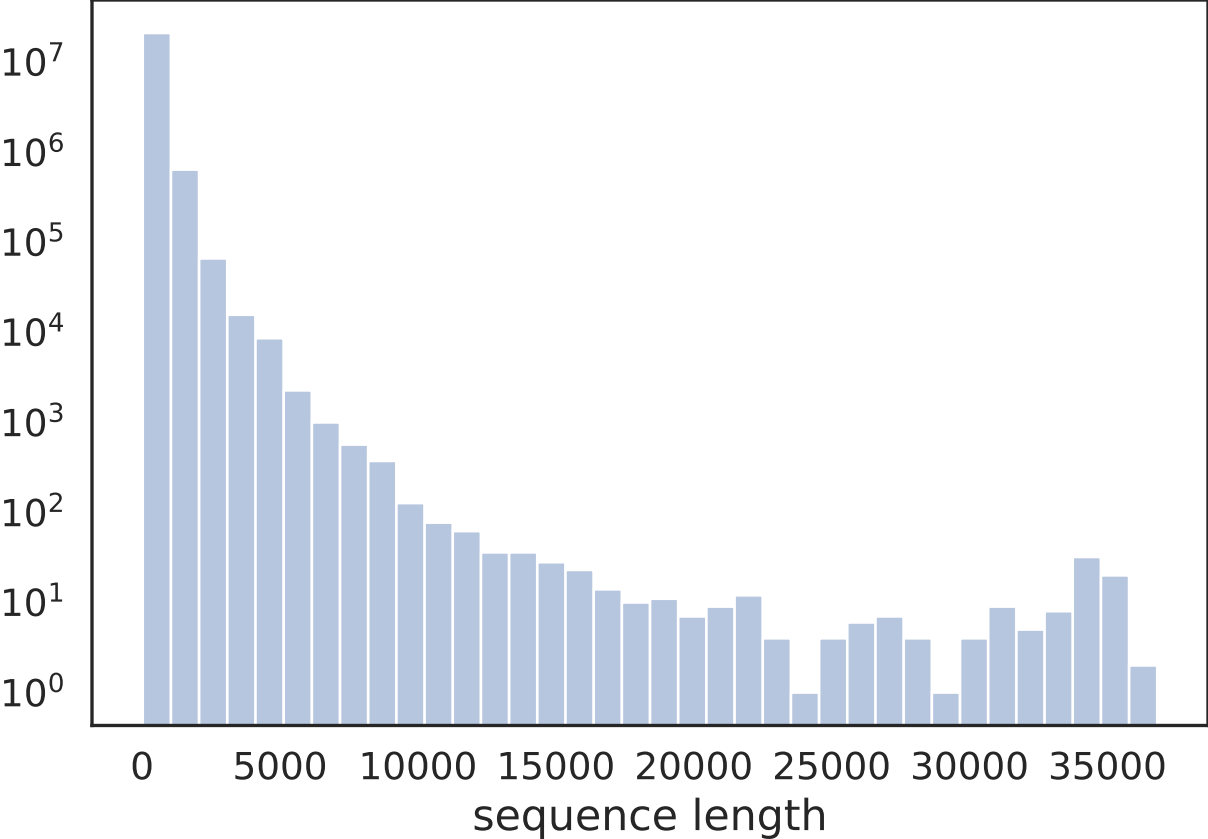

Supplement: btaa1051_Supplementary_Data [file btaa1051_supplementary_data.zip › sequence_lengths_eggNOG5_1.pdf]

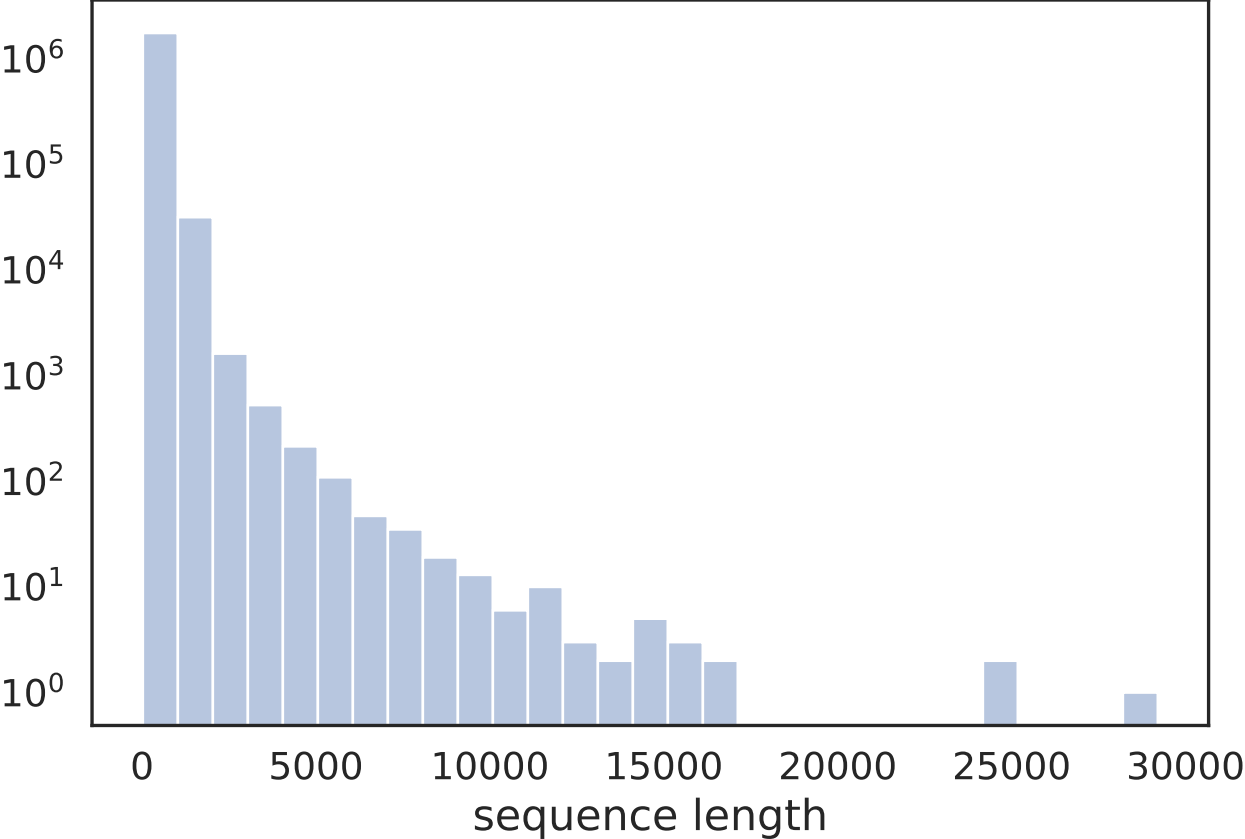

Supplement: btaa1051_Supplementary_Data [file btaa1051_supplementary_data.zip › sequence_lengths_COG_2014.pdf]

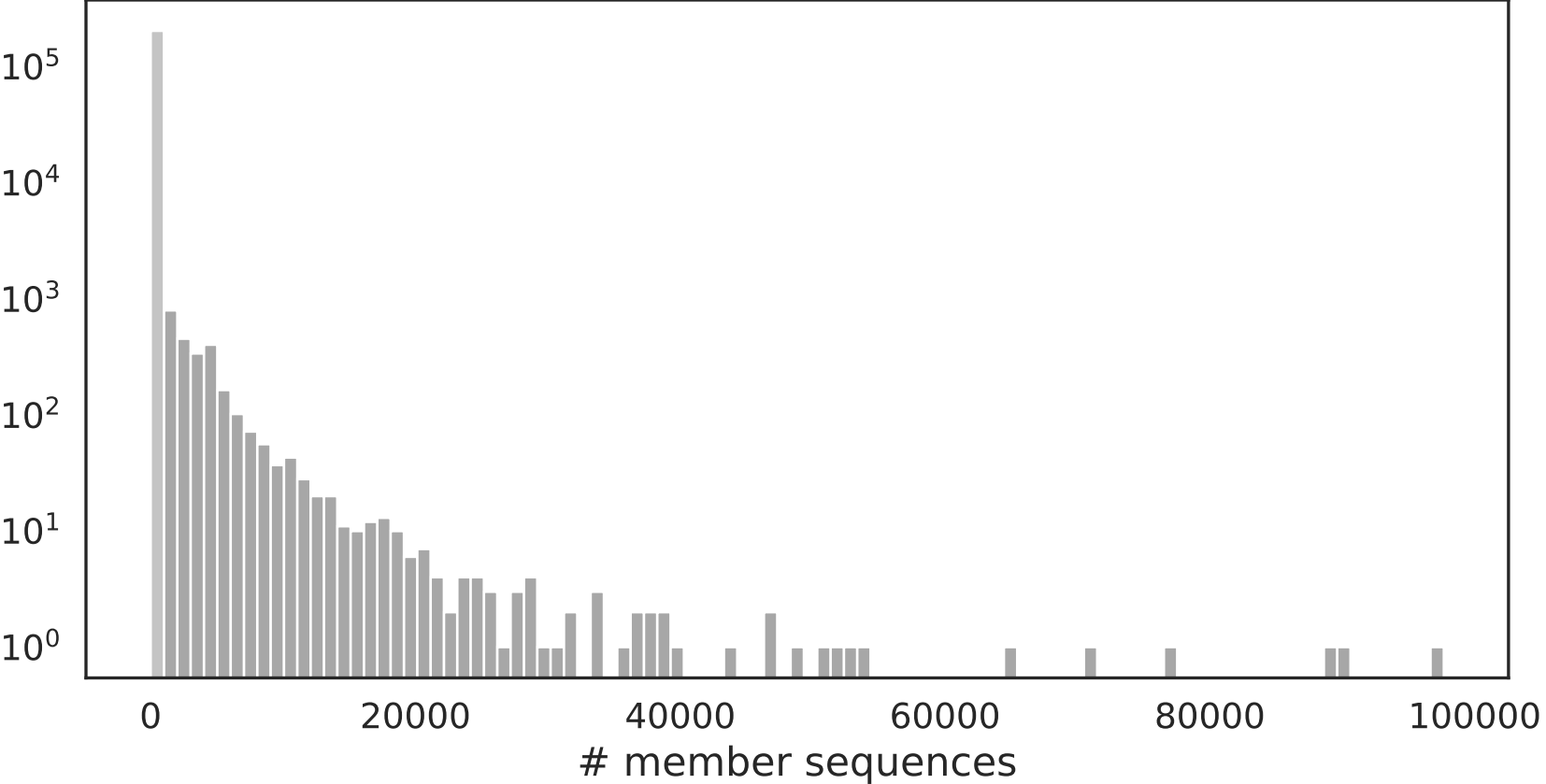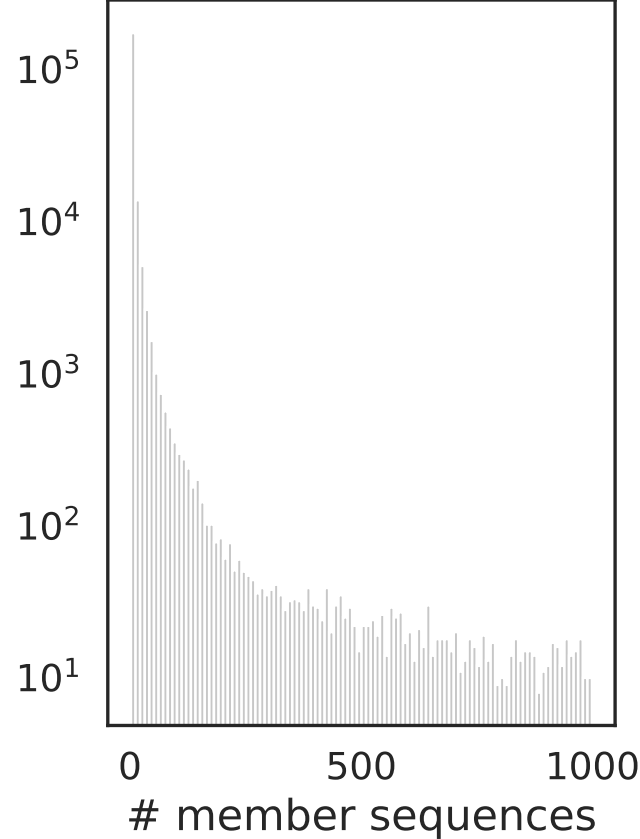

Supplement: btaa1051_Supplementary_Data [file btaa1051_supplementary_data.zip › population_eggNOG5_2.pdf]

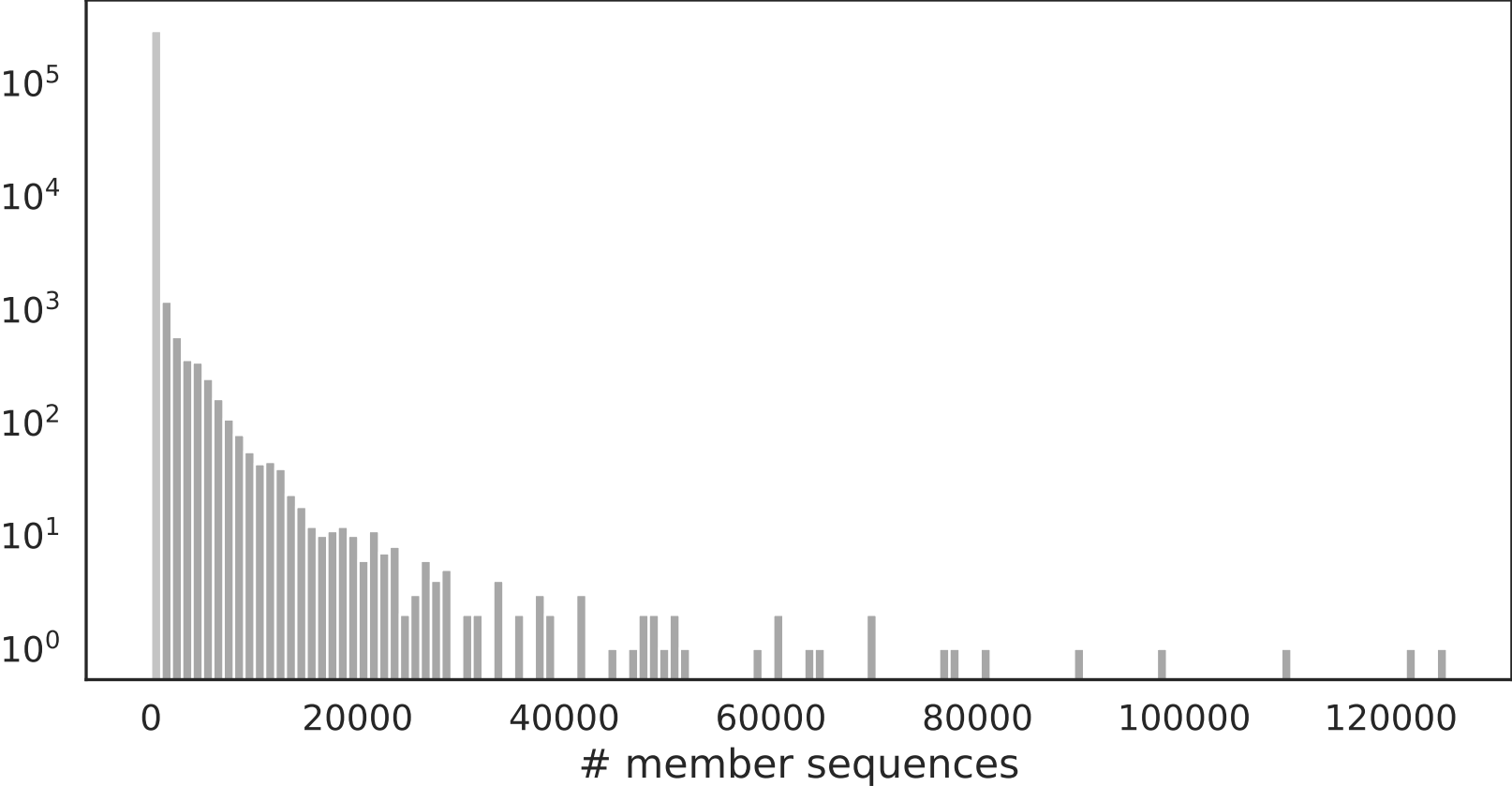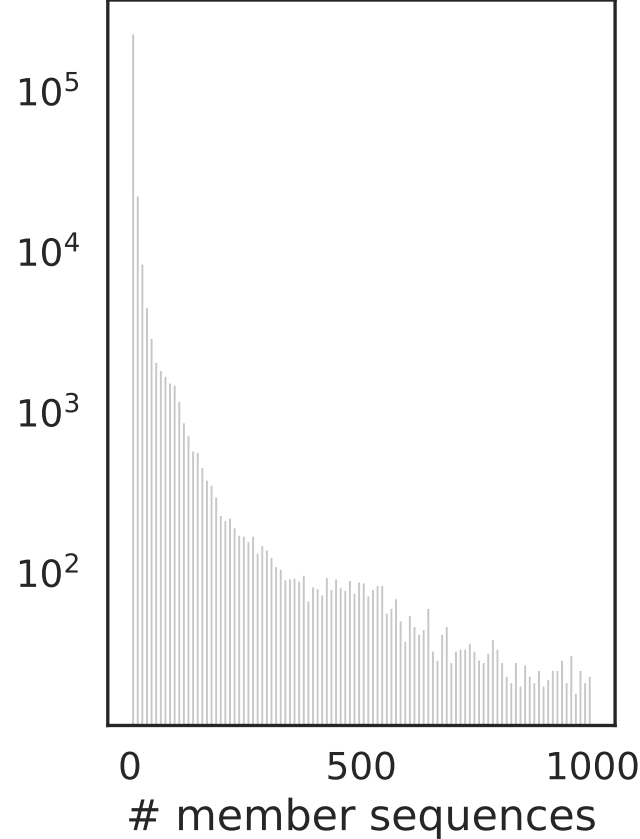

Supplement: btaa1051_Supplementary_Data [file btaa1051_supplementary_data.zip › population_eggNOG5_1.pdf]

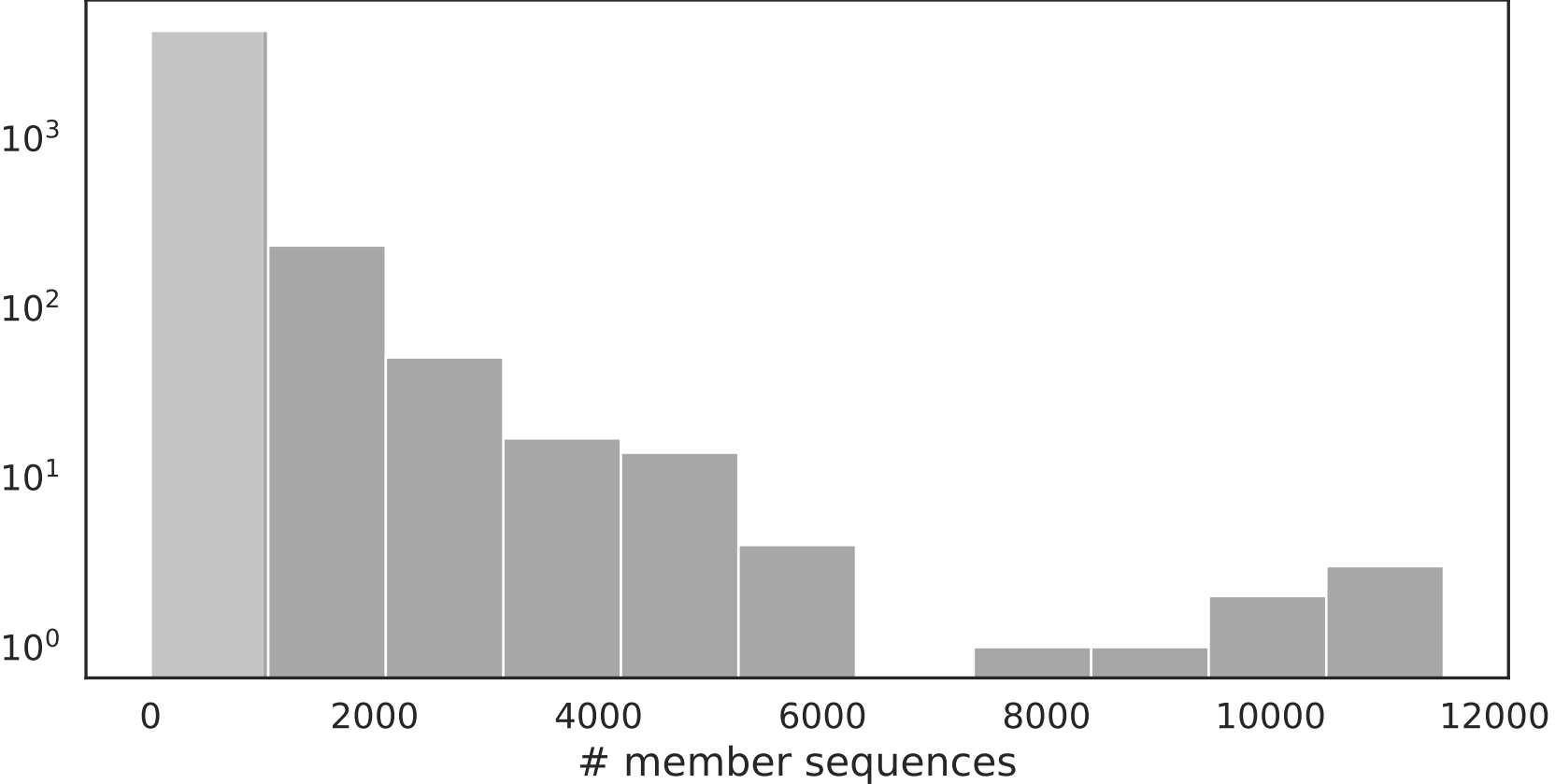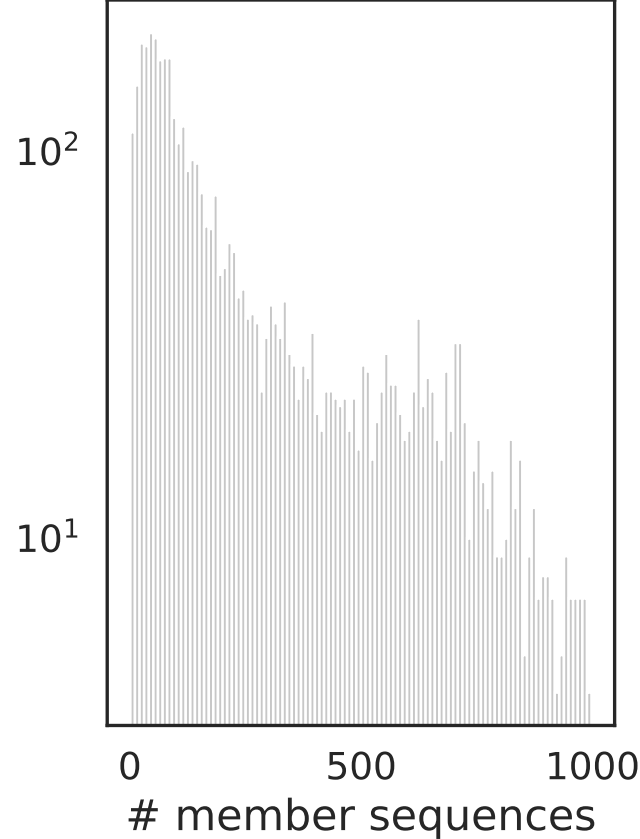

Supplement: btaa1051_Supplementary_Data [file btaa1051_supplementary_data.zip › population_COG_2014.pdf]

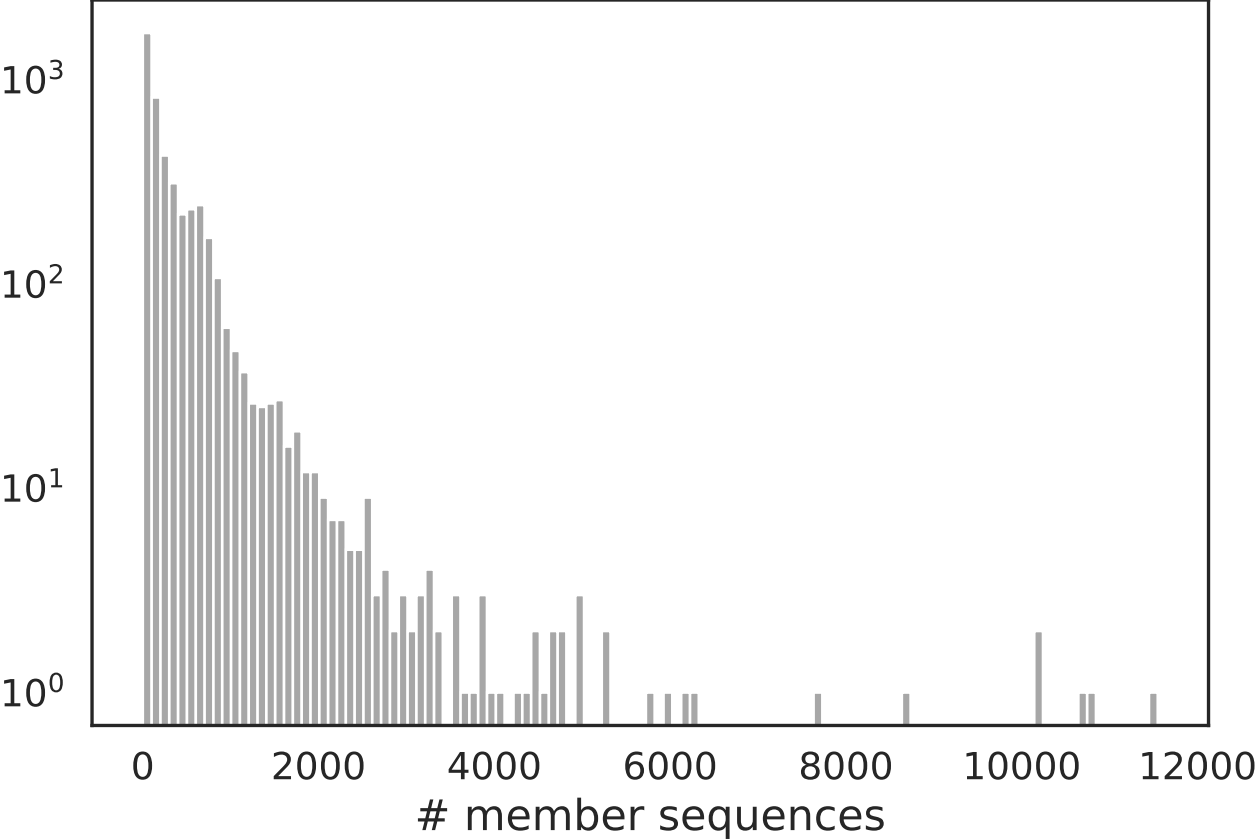

Supplement: btaa1051_Supplementary_Data [file btaa1051_supplementary_data.zip › population_cog.pdf]

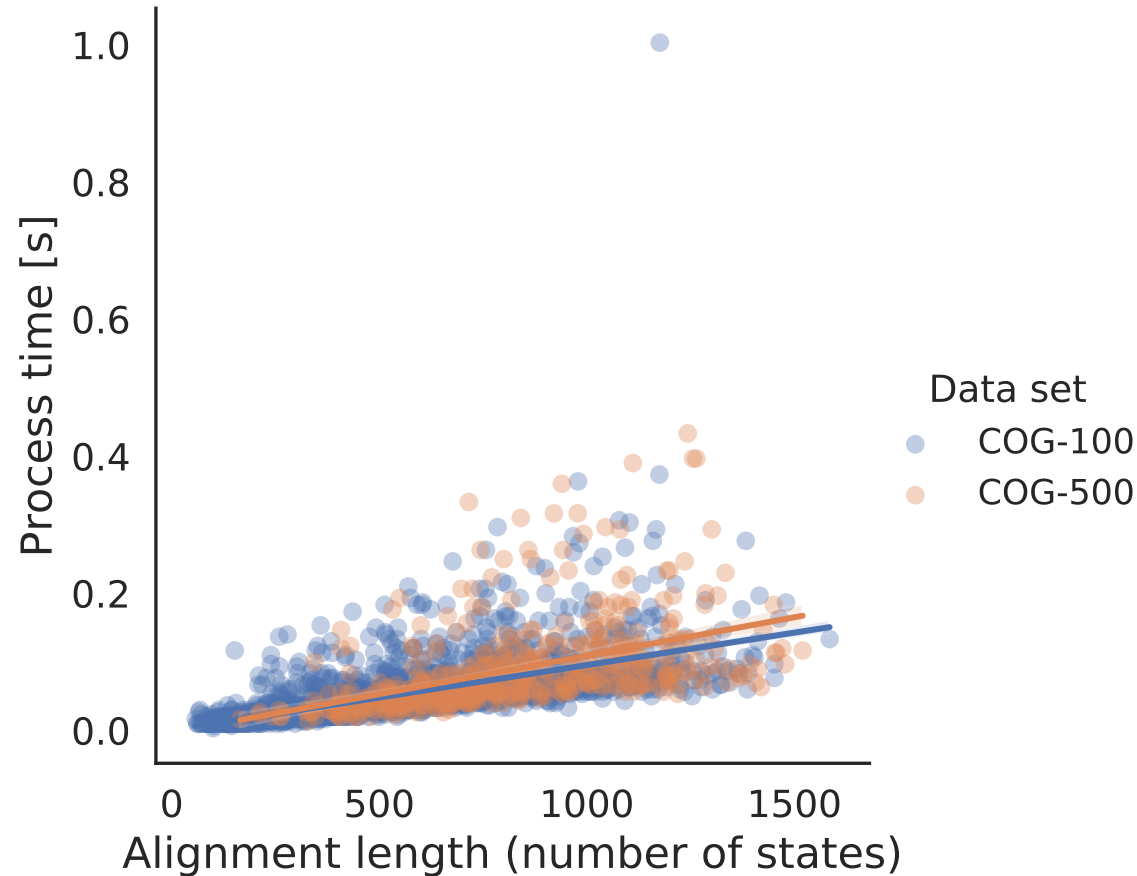

Supplement: btaa1051_Supplementary_Data [file btaa1051_supplementary_data.zip › deepnog_rev2min7_hmmtime.pdf]

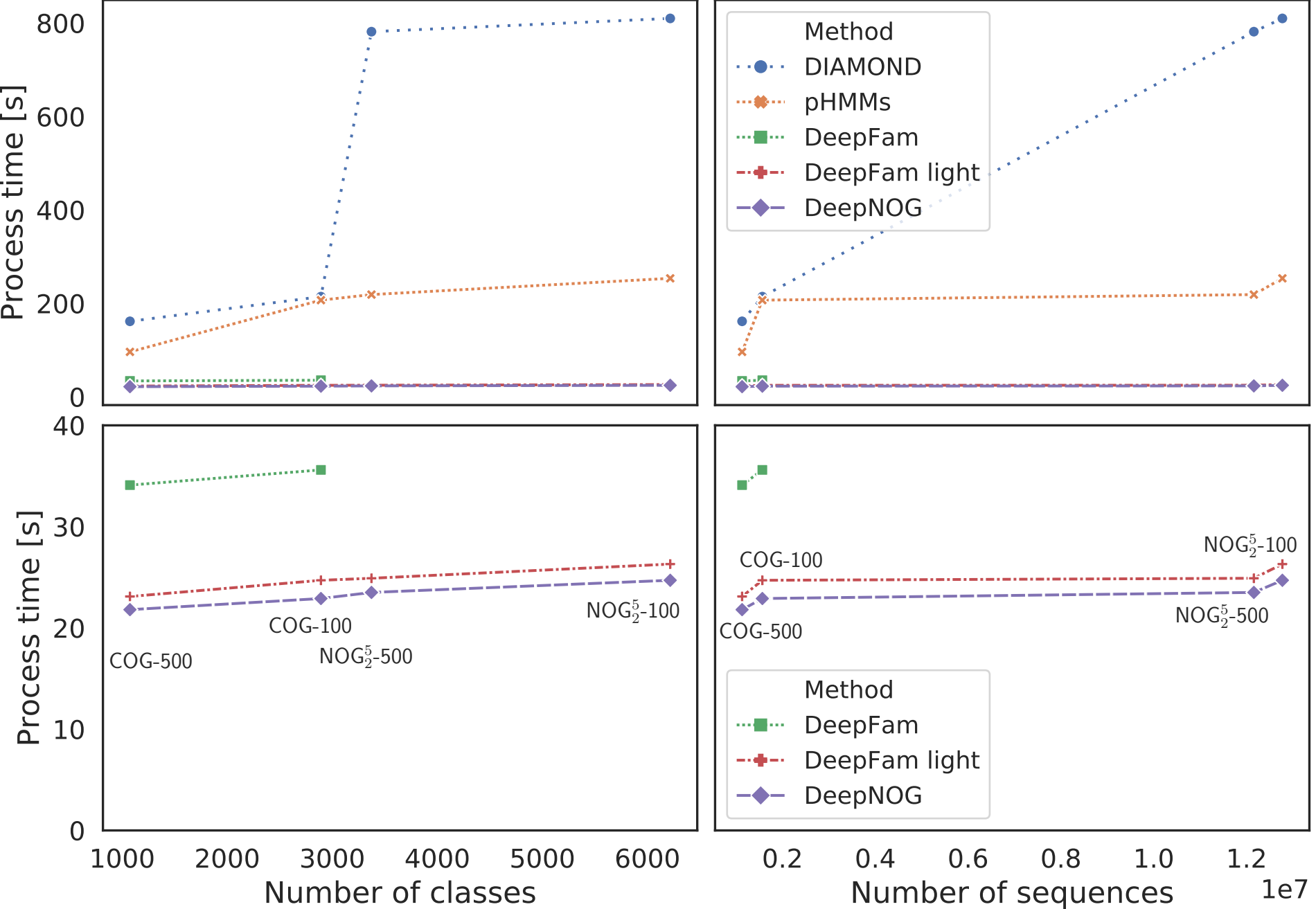

Supplement: btaa1051_Supplementary_Data [file btaa1051_supplementary_data.zip › deepnog_rev2min7.pdf]
